# NASICON-related Na_3.4_Mn_0.4_Fe_1.6_(PO_4_)_3_

**DOI:** 10.1107/S1600536812027584

**Published:** 2012-06-23

**Authors:** Michael M. Yatskin, Nataliya Yu. Strutynska, Vyacheslav N. Baumer, Ivan V. Ogorodnyk, Nikolay S. Slobodyanik

**Affiliations:** a Department of Inorganic Chemistry, Taras Shevchenko National University, 64 Volodymyrska str., 01601 Kyiv, Ukraine; b STC "Institute for Single Crystals", NAS of Ukraine, 60 Lenin ave., 61001 Kharkiv, Ukraine

## Abstract

The solid solution, sodium [iron(III)/manganese(II)] tris­(orthophosphate), Na_3.4_Mn_0.4_Fe_1.6_(PO_4_)_3_, was obtained using a flux method. Its crystal structure is related to that of NASICON-type compounds. The [(Mn/Fe)_2_(PO_4_)_3_] framework is built up from an (Mn/Fe)O_6_ octa­hedron (site symmetry 3.), with a mixed Mn/Fe occupancy, and a PO_4_ tetra­hedron (site symmetry .2). The Na^+^ cations are distributed over two partially occupied sites in the cavities of the framework. One Na^+^ cation (site symmetry -3.) is surrounded by six O atoms, whereas the other Na^+^ cation (site symmetry .2) is surrounded by eight O atoms.

## Related literature

For applications and properties of NASICON-related compounds, see: Goodenough *et al.* (1976 ▶); Shimizu & Ushijima (2000 ▶); Veríssimo *et al.* (1997 ▶); Mariappan *et al.* (2005 ▶); Arbi *et al.* (2002 ▶); Moreno-Real *et al.* (2002 ▶). For details of structural relationships with other compounds, see: γ-Na_3_Fe_2_(PO_4_)_3_ (Masquelier *et al.*, 2000 ▶); Na_4_Fe_2_(PO_4_)_3_ (Hatert, 2009 ▶); Na_4_MgFe(PO_4_)_3_ (Strutynska *et al.*, 2012 ▶); Na_4_NiFe(PO_4_)_3_ (Essehli *et al.*, 2011 ▶).

## Experimental

### 

#### Crystal data

Na_3.4_Mn_0.4_Fe_1.6_(PO_4_)_3_
*M* *_r_* = 474.41
Trigonal, 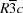

*a* = 8.8694 (2) Å
*c* = 21.6074 (7) Å
*V* = 1472.05 (7) Å^3^
*Z* = 6
Mo *K*α radiation
μ = 3.59 mm^−1^
*T* = 293 K
0.10 × 0.10 × 0.08 mm

#### Data collection

Oxford Diffraction Xcalibur-3 diffractometer
Absorption correction: multi-scan (Blessing, 1995 ▶) *T*_min_ = 0.721, *T*_max_ = 0.795
8867 measured reflections
728 independent reflections
658 reflections with *I* > 2σ(*I*)
*R*_int_ = 0.033

#### Refinement

*R*[*F* ^2^ > 2σ(*F* ^2^)] = 0.028
*wR*(*F* ^2^) = 0.072
*S* = 1.23
728 reflections
38 parameters
2 restraints
Δρ_max_ = 0.66 e Å^−3^
Δρ_min_ = −0.39 e Å^−3^

### 

Data collection: *CrysAlis CCD* (Oxford Diffraction, 2006 ▶); cell refinement: *CrysAlis CCD*; data reduction: *CrysAlis RED* (Oxford Diffraction, 2006 ▶); program(s) used to solve structure: *SHELXS97* (Sheldrick, 2008 ▶); program(s) used to refine structure: *SHELXL97* (Sheldrick, 2008 ▶); molecular graphics: *DIAMOND* (Brandenburg, 1999 ▶); software used to prepare material for publication: *WinGX* (Farrugia, 1999 ▶) and *enCIFer* (Allen *et al.*, 2004 ▶).

## Supplementary Material

Crystal structure: contains datablock(s) global, I. DOI: 10.1107/S1600536812027584/wm2642sup1.cif

Structure factors: contains datablock(s) I. DOI: 10.1107/S1600536812027584/wm2642Isup2.hkl

Additional supplementary materials:  crystallographic information; 3D view; checkCIF report

## Figures and Tables

**Table 1:** Selected bond lengths (Å)

Na1—O2	2.4546 (15)
Na2—O2^i^	2.4505 (16)
Na2—O2^ii^	2.472 (2)
Na2—O1^i^	2.587 (2)
Na2—O1^iii^	2.921 (2)
Fe1—O1	1.9962 (16)
Fe1—O2	2.1053 (15)
P1—O1	1.5244 (16)
P1—O2^ii^	1.5346 (15)

Symmetry codes: (i) 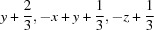; (ii) 
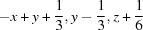
; (iii) 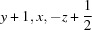.

**Fig. 1.**
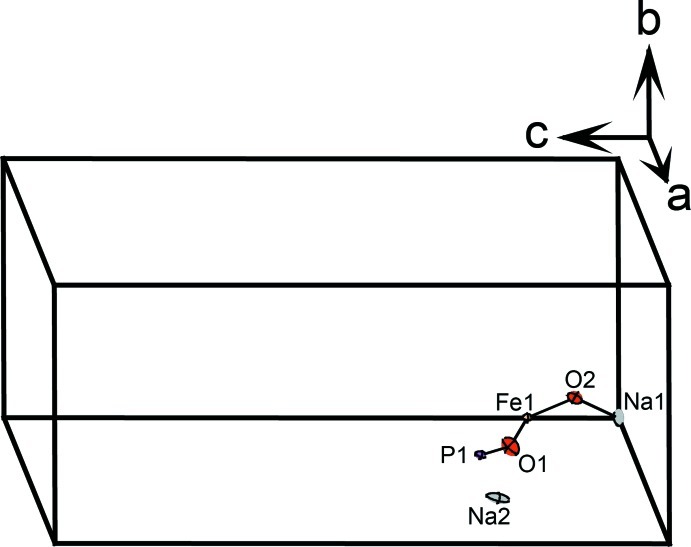
The asymmetric unit of (I), showing displacement ellipsoids at the 50% probability level.

**Fig. 2.**
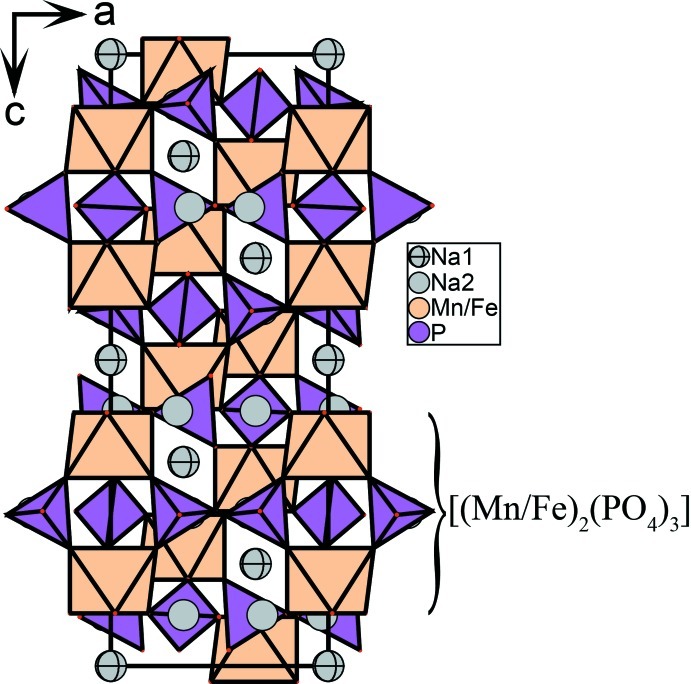
Elementary fragments and three-dimensional framework in the title compound.

## supplementary crystallographic information

### Comment

NASICON-type compounds possess high ionic conductivity, chemical stability and attract great interest for application in solid-state electrochemical devices (Goodenough *et al.*, 1976; Shimizu & Ushijima, 2000; Veríssimo *et al.*, 1997; Mariappan *et al.*, 2005; Arbi *et al.*, 2002; Moreno-Real *et al.*, 2002).

Herein, the structure of Na_3.4_Mn_0.4_Fe_1.6_(PO_4_)_3_, (I), is reported. Compound (I) can be considered as a solid solution of γ-Na_3_Fe_2_(PO_4_)_3_ (Masquelier *et al.*, 2000) and belongs to the NASICON structure type.

There are two Na sites (Wyckoff positions 6*b* and 18*e*), one mixed occupied Mn/Fe site (12*c*), one P site (18*e*) and two O sites (36*f*) in the asymmetric unit of (I) (Fig. 1). The basic building block of the structure is the [(Mn/Fe)_2_(PO_4_)_3_] unit, which consists of two (Mn/Fe)O_6_ polyhedra interlinked by three bridging PO_4_-tetrahedra (Fig. 2). These fragments alternate with Na1O_6_-polyhedra along [001] forming ribbons, which in turn are interconnected by PO_4_-tetrahedra forming a three-dimensional framework (Fig. 2). The distances *M*—O in the (Mn/Fe)O_6_ octahedra vary from 1.9962 (16) to 2.1053 (15) Å and are similar to that in isotypic structures (*e.g.* 1.956 (2)–2.048 (2) Å in γ-Na_3_Fe_2_(PO_4_)_3_ (Masquelier *et al.*, 2000); 2.010 (6)–2.130 (6) Å in Na_4_Fe_2_(PO_4_)_3_ (Hatert, 2009); 1.926 (5)–2.037 (6) Å in Na_4_MgFe(PO_4_)_3_ (Strutynska *et al.*, 2012); 1.955 (3)–2.050 (3) Å in Na_4_NiFe(PO_4_)_3_ (Essehli *et al.*, 2011). The P atom has an almost regular tetrahedral coordination, the P—O distances in the PO_4_ tetrahedra being in the range 1.5244 (16)–1.5346 (15) Å, as is typically observed in NASICON-type phosphates. Two types of sodium atoms occupy the cavities of the framework. The Na1 atoms (s.o.f. = 0.848 (5)) lie on a threefold roto-inversion axis and are surrounded by six O2 atoms in a distance of 2.4546 (15) Å. The Na2 (s.o.f. = 0.853 (5)) coordination environment is formed by eight oxygen atoms with four pairs of equal contacts (*d*(Na2—O) = 2.4505 (16)–2.921 (2) Å, using a cut-off distance of 3.1 Å).

### Experimental

The title compound was obtained during investigation of the melting system Na_2_O–P_2_O_5_–Fe_2_O_3_–MnO. A mixture of NaPO_3_ (12.24 g), Na_2_CO_3_ (1.908 g), Fe_2_O_3_ (2.4 g) and MnCO_3_^.^Mn(OH)_2_ (3.8 g) was ground in an agate mortar, placed in a platinum crucible and heated up to 1273 K. The melt was kept at this temperature for 3 h. After that, the temperature was cooled down to 973 K at a rate of 10 K/h. The light-violet crystals of (I) were recovered using hot water. The chemical composition of single-crystal was verified using EDX analysis. Analysis found: Na 16.62, Mn 2.14, Fe 7.93, P 14.94 and O 58.37 at%, while Na_3.4_Mn_0.4_Fe_1.6_(PO_4_)_3_ requires Na 16.67, Mn 1.96, Fe 7.84, P 14.70 and O 58.82 at%.

### Refinement

For refinement of the Fe/Mn ratio and the Na-content, SUMP instructions in *SHELXL* (Sheldrick, 2008) were employed, assuming full occupancy of the (Fe/Mn) site and an average charge of the (Fe/Mn) and Na sites of +9. The refined composition is close to that determined by EDX measurements. The highest remaining peak in the final difference Fourier map is 0.76 A from P1 and the deepest hole is 1.09 Å from the same atom.

### Crystal data

**Table:**

Na_3.4_Mn_0.4_Fe_1.6_(PO_4_)_3_	*D*_x_ = 3.212 Mg m^−^^3^
*M**_r_* = 474.41	Mo *K*α radiation, λ = 0.71073 Å
Trigonal, *R*3*c*	Cell parameters from 8867 reflections
Hall symbol: -R 3 2" c	θ = 3.3–35.0°
*a* = 8.8694 (2) Å	µ = 3.59 mm^−^^1^
*c* = 21.6074 (7) Å	*T* = 293 K
*V* = 1472.05 (7) Å^3^	Prism, light-violet
*Z* = 6	0.10 × 0.10 × 0.08 mm
*F*(000) = 1380	

### Data collection

**Table:**

Oxford Diffraction Xcalibur-3 diffractometer	728 independent reflections
Radiation source: fine-focus sealed tube	658 reflections with *I* > 2σ(*I*)
Graphite monochromator	*R*_int_ = 0.033
φ and ω scans	θ_max_ = 35.0°, θ_min_ = 3.3°
Absorption correction: multi-scan (Blessing, 1995)	*h* = −14→14
*T*_min_ = 0.721, *T*_max_ = 0.795	*k* = −13→14
8867 measured reflections	*l* = −34→34

### Refinement

**Table:**

Refinement on *F*^2^	2 restraints
Least-squares matrix: full	Primary atom site location: structure-invariant direct methods
*R*[*F*^2^ > 2σ(*F*^2^)] = 0.028	Secondary atom site location: difference Fourier map
*wR*(*F*^2^) = 0.072	*w* = 1/[σ^2^(*F*_o_^2^) + (0.0324*P*)^2^ + 3.4282*P*] where *P* = (*F*_o_^2^ + 2*F*_c_^2^)/3
*S* = 1.23	(Δ/σ)_max_ = 0.064
728 reflections	Δρ_max_ = 0.66 e Å^−^^3^
38 parameters	Δρ_min_ = −0.39 e Å^−^^3^

### Special details

**Table:**

Geometry. All e.s.d.'s (except the e.s.d. in the dihedral angle between two l.s. planes) are estimated using the full covariance matrix. The cell e.s.d.'s are taken into account individually in the estimation of e.s.d.'s in distances, angles and torsion angles; correlations between e.s.d.'s in cell parameters are only used when they are defined by crystal symmetry. An approximate (isotropic) treatment of cell e.s.d.'s is used for estimating e.s.d.'s involving l.s. planes.
Refinement. Refinement of *F*^2^ against ALL reflections. The weighted *R*-factor *wR* and goodness of fit *S* are based on *F*^2^, conventional *R*-factors *R* are based on *F*, with *F* set to zero for negative *F*^2^. The threshold expression of *F*^2^ > σ(*F*^2^) is used only for calculating *R*-factors(gt) *etc*. and is not relevant to the choice of reflections for refinement. *R*-factors based on *F*^2^ are statistically about twice as large as those based on *F*, and *R*- factors based on ALL data will be even larger.

### Fractional atomic coordinates and isotropic or equivalent isotropic displacement parameters (Å^2^)

**Table:**

	*x*	*y*	*z*	*U*_iso_*/*U*_eq_	Occ. (<1)
Na1	0	0	0	0.0322 (8)	0.848 (5)
Na2	0.63677 (18)	0	0.25	0.0356 (6)	0.853 (5)
Fe1	0	0	0.149480 (18)	0.01045 (11)	0.7991 (12)
Mn1	0	0	0.149480 (18)	0.01045 (11)	0.2009 (12)
P1	0.29637 (7)	0	0.25	0.01144 (13)	
O1	0.1887 (2)	−0.0197 (3)	0.19242 (8)	0.0346 (4)	
O2	0.18883 (19)	0.17055 (18)	0.08615 (7)	0.0214 (3)	

### Atomic displacement parameters (Å^2^)

**Table:**

	*U*^11^	*U*^22^	*U*^33^	*U*^12^	*U*^13^	*U*^23^
Na1	0.0421 (11)	0.0421 (11)	0.0124 (10)	0.0211 (6)	0	0
Na2	0.0206 (6)	0.0151 (7)	0.0694 (14)	0.0075 (3)	0.0068 (4)	0.0136 (7)
Fe1	0.01042 (13)	0.01042 (13)	0.01049 (17)	0.00521 (7)	0	0
Mn1	0.01042 (13)	0.01042 (13)	0.01049 (17)	0.00521 (7)	0	0
P1	0.01059 (19)	0.0092 (2)	0.0141 (2)	0.00459 (12)	0.00093 (9)	0.00185 (18)
O1	0.0270 (8)	0.0404 (10)	0.0327 (8)	0.0142 (7)	−0.0155 (7)	0.0016 (7)
O2	0.0157 (6)	0.0139 (6)	0.0274 (7)	0.0021 (5)	−0.0002 (5)	0.0021 (5)

### Geometric parameters (Å, º)

**Table:**

Na1—O2^i^	2.4546 (15)	Na2—O1^x^	2.921 (2)
Na1—O2	2.4546 (15)	Na2—O1^xi^	2.921 (2)
Na1—O2^ii^	2.4546 (15)	Fe1—O1^ii^	1.9962 (16)
Na1—O2^iii^	2.4546 (15)	Fe1—O1^iii^	1.9962 (16)
Na1—O2^iv^	2.4546 (15)	Fe1—O1	1.9962 (16)
Na1—O2^v^	2.4546 (15)	Fe1—O2^ii^	2.1053 (15)
Na2—O2^vi^	2.4505 (16)	Fe1—O2^iii^	2.1053 (15)
Na2—O2^vii^	2.4505 (16)	Fe1—O2	2.1053 (15)
Na2—O2^viii^	2.472 (2)	P1—O1	1.5244 (16)
Na2—O2^ix^	2.472 (2)	P1—O1^xii^	1.5244 (16)
Na2—O1^vii^	2.587 (2)	P1—O2^ix^	1.5346 (15)
Na2—O1^vi^	2.587 (2)	P1—O2^viii^	1.5346 (15)
			
O2^i^—Na1—O2	180.00 (5)	O1^vii^—Na2—O1^xi^	109.85 (7)
O2^i^—Na1—O2^ii^	111.27 (5)	O1^vi^—Na2—O1^xi^	86.08 (4)
O2—Na1—O2^ii^	68.73 (5)	O1^x^—Na2—O1^xi^	81.67 (9)
O2^i^—Na1—O2^iii^	111.27 (5)	O1^ii^—Fe1—O1^iii^	100.14 (7)
O2—Na1—O2^iii^	68.73 (5)	O1^ii^—Fe1—O1	100.14 (7)
O2^ii^—Na1—O2^iii^	68.73 (5)	O1^iii^—Fe1—O1	100.14 (7)
O2^i^—Na1—O2^iv^	68.73 (5)	O1^ii^—Fe1—O2^ii^	87.95 (7)
O2—Na1—O2^iv^	111.27 (5)	O1^iii^—Fe1—O2^ii^	167.15 (7)
O2^ii^—Na1—O2^iv^	111.27 (5)	O1—Fe1—O2^ii^	88.10 (7)
O2^iii^—Na1—O2^iv^	180.00 (8)	O1^ii^—Fe1—O2^iii^	88.10 (7)
O2^i^—Na1—O2^v^	68.73 (5)	O1^iii^—Fe1—O2^iii^	87.95 (7)
O2—Na1—O2^v^	111.27 (5)	O1—Fe1—O2^iii^	167.15 (7)
O2^ii^—Na1—O2^v^	180.00 (9)	O2^ii^—Fe1—O2^iii^	82.32 (6)
O2^iii^—Na1—O2^v^	111.27 (5)	O1^ii^—Fe1—O2	167.15 (7)
O2^iv^—Na1—O2^v^	68.73 (5)	O1^iii^—Fe1—O2	88.10 (7)
O2^vi^—Na2—O2^vii^	162.12 (10)	O1—Fe1—O2	87.95 (7)
O2^vi^—Na2—O2^viii^	129.35 (6)	O2^ii^—Fe1—O2	82.32 (6)
O2^vii^—Na2—O2^viii^	68.52 (7)	O2^iii^—Fe1—O2	82.32 (6)
O2^vi^—Na2—O2^ix^	68.52 (7)	O1—P1—O1^xii^	110.62 (16)
O2^vii^—Na2—O2^ix^	129.35 (6)	O1—P1—O2^ix^	112.19 (10)
O2^viii^—Na2—O2^ix^	60.85 (8)	O1^xii^—P1—O2^ix^	106.30 (9)
O2^vi^—Na2—O1^vii^	114.64 (5)	O1—P1—O2^viii^	106.30 (9)
O2^vii^—Na2—O1^vii^	68.83 (5)	O1^xii^—P1—O2^viii^	112.19 (10)
O2^viii^—Na2—O1^vii^	68.62 (6)	O2^ix^—P1—O2^viii^	109.33 (12)
O2^ix^—Na2—O1^vii^	93.19 (7)	P1—O1—Fe1	150.92 (13)
O2^vi^—Na2—O1^vi^	68.83 (5)	P1—O1—Na2^xiii^	120.86 (11)
O2^vii^—Na2—O1^vi^	114.64 (5)	Fe1—O1—Na2^xiii^	86.63 (6)
O2^viii^—Na2—O1^vi^	93.19 (7)	P1—O1—Na2^xiv^	76.78 (7)
O2^ix^—Na2—O1^vi^	68.62 (6)	Fe1—O1—Na2^xiv^	102.77 (7)
O1^vii^—Na2—O1^vi^	159.36 (11)	Na2^xiii^—O1—Na2^xiv^	112.45 (8)
O2^vi^—Na2—O1^x^	53.35 (5)	P1^ix^—O2—Fe1	141.73 (9)
O2^vii^—Na2—O1^x^	111.21 (7)	P1^ix^—O2—Na2^xiii^	93.51 (8)
O2^viii^—Na2—O1^x^	153.24 (6)	Fe1—O2—Na2^xiii^	87.96 (6)
O2^ix^—Na2—O1^x^	114.32 (5)	P1^ix^—O2—Na1	128.40 (8)
O1^vii^—Na2—O1^x^	86.07 (4)	Fe1—O2—Na1	89.86 (5)
O1^vi^—Na2—O1^x^	109.85 (7)	Na2^xiii^—O2—Na1	86.38 (5)
O2^vi^—Na2—O1^xi^	111.22 (7)	P1^ix^—O2—Na2^ix^	94.91 (7)
O2^vii^—Na2—O1^xi^	53.35 (5)	Fe1—O2—Na2^ix^	87.39 (6)
O2^viii^—Na2—O1^xi^	114.32 (5)	Na2^xiii^—O2—Na2^ix^	171.00 (7)
O2^ix^—Na2—O1^xi^	153.24 (6)	Na1—O2—Na2^ix^	85.91 (5)

Symmetry codes: (i) −*x*, −*y*, −*z*; (ii) −*x*+*y*, −*x*, *z*; (iii) −*y*, *x*−*y*, *z*; (iv) *y*, −*x*+*y*, −*z*; (v) *x*−*y*, *x*, −*z*; (vi) *y*+2/3, −*x*+*y*+1/3, −*z*+1/3; (vii) *x*+1/3, *x*−*y*−1/3, *z*+1/6; (viii) −*x*+*y*+1/3, *y*−1/3, *z*+1/6; (ix) −*x*+2/3, −*y*+1/3, −*z*+1/3; (x) *y*+1, *x*, −*z*+1/2; (xi) −*x*+*y*+1, −*x*, *z*; (xii) *x*−*y*, −*y*, −*z*+1/2; (xiii) *x*−*y*−1/3, *x*−2/3, −*z*+1/3; (xiv) −*y*, *x*−*y*−1, *z*.
